# Extracellular vesicles in disorders of hemostasis following traumatic brain injury

**DOI:** 10.3389/fneur.2024.1373266

**Published:** 2024-05-09

**Authors:** Aisling Mc Mahon, Luisa Weiss, Kathleen Bennett, Ger Curley, Fionnuala Ní Ainle, Patricia Maguire

**Affiliations:** ^1^School of Biomolecular and Biomedical Science, University College Dublin, Dublin, Ireland; ^2^Department of Critical Care Medicine, Mater Misericordiae University Hospital, Dublin, Ireland; ^3^SPHERE Research Group, Conway Institute, University College Dublin, Dublin, Ireland; ^4^Data Science Centre, School of Population Health, RCSI University of Medicine and Health Sciences, Dublin, Ireland; ^5^Department of Anaesthesia and Critical Care Medicine, Beaumont Hospital, Dublin, Ireland; ^6^Department of Haematology, Mater Misericordiae University Hospital and Rotunda Hospital, School of Medicine, University College Dublin, Dublin, Ireland; ^7^UCD Institute for Discovery, O'Brien Centre for Science, Dublin, Ireland

**Keywords:** traumatic brain injury, extracellular vesicles, brain-derived extracellular vesicles, haemostasis, thrombosis, biomarkers

## Abstract

Traumatic brain injury (TBI) is a global health priority. In addition to being the leading cause of trauma related death, TBI can result in long-term disability and loss of health. Disorders of haemostasis are common despite the absence of some of the traditional risk factors for coagulopathy following trauma. Similar to trauma induced coagulopathy, this manifests with a biphasic response consisting of an early hypocoagulable phase and delayed hypercoagulable state. This coagulopathy is clinically significant and associated with increased rates of haemorrhagic expansion, disability and death. The pathophysiology of TBI-induced coagulopathy is complex but there is biologic plausibility and emerging evidence to suggest that extracellular vesicles (EVs) have a role to play. TBI and damage to the blood brain barrier result in release of brain-derived EVs that contain tissue factor and phosphatidylserine on their surface. This provides a platform on which coagulation can occur. Preclinical animal models have shown that an early rapid release of EVs results in overwhelming activation of coagulation resulting in a consumptive coagulopathy. This phenomenon can be attenuated with administration of substances to promote EV clearance and block their effects. Small clinical studies have demonstrated elevated levels of procoagulant EVs in patients with TBI correlating with clinical outcome. EVs represent a promising opportunity for use as minimally invasive biomarkers and potential therapeutic targets for TBI patients. However, additional research is necessary to bridge the gap between their potential and practical application in clinical settings.

## Introduction

Traumatic Brain Injury (TBI) has been recognized as a global health priority. In 2016, there were more than 27 million cases of TBI worldwide. Between 1990 and 2016, the annual incidence increased by 3.6% (1). This increase is in part explained by an aging population, with falls being the most common cause of TBI. Despite road safety initiatives, a substantial proportion of TBI occur after motor vehicle accidents as population density and use of motor vehicles rises. In addition to being the leading cause of trauma related mortality, TBI leads to long-term disability and loss of health with an estimated 8.1 million years of life lived with disability following TBI in 2016 alone (1). In addition to this, TBI places a significant burden on healthcare systems with economic implications associated with care required for these patients. There are also more widespread societal costs resulting from loss of individuals from the workplace.

Following trauma, around one quarter of patients will develop a trauma-induced coagulopathy (TIC), defined as a prothrombin ratio of 1.2 or higher and an activated partial thromboplastin time greater than 60 (2). This typically manifests a biphasic response, with an early hypocoagulable state and a later hypercoagulable profile (3). It has been recognized that there is a unique coagulopathy phenotype in TBI independent of TIC, diagnosed with either conventional clotting assays (CCAs) or viscoelastic assays (VEAs) (4). This is despite the fact that isolated TBI often lacks some of the key causal factors of coagulopathy in general trauma patients, namely significant blood loss and hemodilution following fluid resuscitation. It is difficult to determine the prevalence of this because of variations in definition but it is estimated that up to two thirds of severe TBI patients will have some form of hemostatic disturbance. The presence of coagulopathy portends a worse outcome with increased rates of primary hemorrhagic transformation, neurosurgical intervention and death. Consistent with TIC, it is thought that there is an early hypocoagulable phase which progresses to a prothrombotic state in the subacute phase after injury (5, 6).

### Pathophysiology of TBI-induced coagulopathy

The pathophysiology behind the coagulopathy of TBI is complex. Direct vascular damage at the time of injury leads to dysfunction of the blood brain barrier (BBB) and this is thought to be integral to the development of coagulopathy. Brain tissue factor (TF) is not normally exposed to the systemic circulation. Following vascular injury and BBB disruption, brain TF may be released resulting in activation of the extrinsic pathway of coagulation, microvascular thrombosis, consumption of clotting factors and a consumptive coagulopathy (5). BBB and microvascular injury also expose platelets to the subendothelial matrix facilitating platelet adhesion either directly or via ligands such as von Willebrand Factor (vWF) leading to formation of a platelet plug. High levels of plasma vWF have been seen in TBI patients and correlate with mortality (7). Brain-derived platelet activating factor (PAF), which may contribute to hypoxia induced BBB dysfunction, further promotes platelet aggregation and platelet hyperactivity is seen in TBI despite reduced platelet counts, resulting in intravascular microthrombosis (5). Platelet hyperactivity and consumption may lead to platelet depletion and eventually platelet exhaustion with increased risk of bleeding. Platelet dysfunction, indicated by increased inhibition of arachidonic acid (AA) and adenosine diphosphate receptors, has been observed in TBI and its mechanism is independent to that of shock or multisystem trauma. Following this initial stage of platelet inhibition and depletion, platelet numbers increase to normal and platelet hyperactivity has been observed at 7 days post TBI with an increase in TEG maximum amplitude (MA) on VEAs (6). This could contribute to the increased venous thromboembolism (VTE) risk seen in TBI patients. Another pathophysiological mechanism implicated in the coagulopathy of TBI is alteration to the fibrinolytic pathway, with three distinct patterns seen post trauma – hyperfibrinolysis, physiologic fibrinolysis and fibrinolytic shutdown. Mortality follows a U-shape curve in line with these groups with early mortality secondary to exsanguination associated with hyperfibrinolysis, late mortality secondary to multiorgan failure (MOF) in the fibrinolytic shutdown group and the nadir in the physiologic group (8). Persistent shutdown at one week it is associated with increased mortality. When looking at TBI patients, it has been shown that fibrinolytic shutdown is seen more frequently than in the overall trauma population, suggesting that coagulation is adapted toward a more hypercoagulable state (9, 10).

### Extracellular vesicles

More recently, attention has been directed toward extracellular vesicles (EVs) and their role in the coagulopathy of TBI. EVs are nano-meter sized particles released from virtually all cells and are surrounded by a lipid bilayer (11). They contain a cargo which consists of lipids, proteins and nucleic acids, both RNA and DNA, that is dependent on their parental cell of origin. EVs are released from the cell via one of two mechanisms, either through fusion of a multivesicular body with the cell membrane and release of its contents, or via cell membrane budding. A considerable proportion of the literature referring to EVs has classified them into exosomes, microparticles, and apoptotic bodies based on size, morphology and composition, acknowledging that there is substantial overlap between subtypes (12). The International Society for Extracellular Vesicles (ISEV) released guidelines, most recently updated in 2018, outlining the minimal information for studies of extracellular vesicles (MISEV). In this they recommend classifying EV subtypes based on operational terms that refer to physical characteristics (e.g., size), biochemical composition, descriptions of conditions or cell of origin rather than using the categorization into exosomes and microparticles (11). EVs have potential for therapeutic use as biomarkers, drug delivery devices and in tissue repair/ regeneration. Their long half-life, low immunogenicity, ability to penetrate cell membranes and potential to target specific cell types make them particularly attractive. From a neurological perspective, the ability to cross the BBB in both directions is advantageous (12, 13).

**Figure 1:**
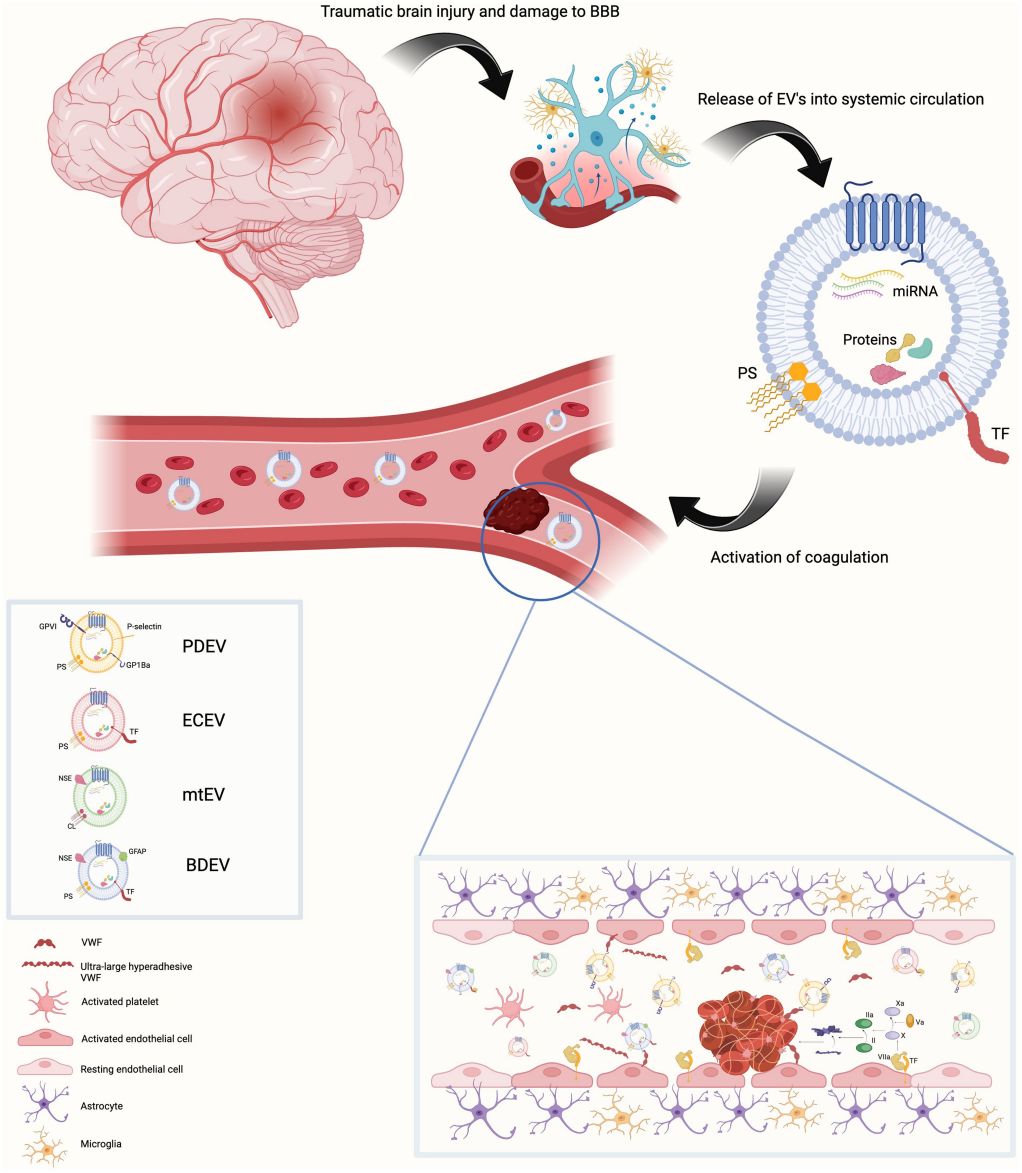
Traumatic brain injury results in damage to the blood brain barrier. This is associated with endothelial cell activation, increased permeability of the blood brain barrier and release of extracellular vesicles (EVs) into blood from a variety of parental cells of origin. These EVs contain the anionic phospholipid phosphatidylserine on their surface (cardiolipin in the case of mitochondrial EVs).Endothelial cell and brain-derived EVs are rich in tissue factor. This provides a platform on which the activation and propagation of coagulation via the extrinsic pathway can occur both within the cerebral vasculature and systemic blood vessels. Activated platelets release EV-bound von Willebrand factor (VWF). EV-bound VWF can also be of neuronal origin. This EV-bound VWF activates endothelial cells resulting in release of ultra-large hyper adhesive VWF. This contributes to the coagulopathy and increases the leakiness of the blood brain barrier. Created with BioRender.com BBB Blood brain barrier, BDEV brain-derived extracellular vesicle, CL cardiolipin, ECEV endothelial cell – derived extracellular vesicle, EV extracellular vesicle, GFAP Glial fibrillary acidic protein, mtEV mitochondrial extracellular vesicle, NSE neuron specific enolase, PDEV platelet-derived extracellular vesicle, PS phosphatidylserine, TF Tissue factor, VWF Von Willebrand factor.

EVs take part in several physiological processes including intercellular communication, cell maintenance, tissue repair and regeneration and the immune response. They also participate in pathological processes and advance disease progression. One of their most well illustrated roles is their contribution to hemostasis, both in health and disease (12–14). It is thought that EVs promote clotting because they provide a surface on which coagulation can occur. In the intact cell, membrane lipids are distributed asymmetrically, with anionic phospholipids such as phosphatidylserine (PS), located on the inner membrane (11, 12, 15). This orientation is reversed in EVs meaning PS can interact with clotting factors and facilitate coagulation. This occurs because of an electrostatic interaction between positively charged γ-carboxyglutamic acid on Factors VII, IX and X and prothrombin and the negatively charged PS (15). Platelet-derived EVs (PDEVs) are known to contain PS on their outer membrane enhancing their procoagulant activity. They also contain receptors for both collagen and von Willebrand factor (VWF). The majority of EVs circulating in blood are believed to be of platelet origin with others originating from erythrocytes, granulocytes and endothelial cells, although this is by no means an exhaustive list and it is not only PDEVs that contain PS (16). EVs may also express tissue factor (TF) on their surface which considerably increases their procoagulant activity. TF expressing EVs have been shown to be involved in cancer associated thrombosis (CAT) (17). Monocyte-derived EVs (MDEVs) are known to contain TF and potentially those shed from neutrophils and endothelial cells but identifying the cellular source of EVs is complex and it is likely that several EV populations are involved in different stages of coagulation simultaneously (12, 14, 15). The procoagulant activity of EVs from different cellular sources may vary due to posttranslational modifications (15). There is a synergistic relationship between TF and PS on EVs. TF requires the presence of anionic phospholipids such as PS to be activated and EVs provide a platform on which this can occur (17). Brain tissue is replete with PS and neuronal and glial cells express TF (Figure 1).

In addition to their procoagulant properties, EVs also have anticoagulant activity. Endothelial cell-derived EVs (ECEVs) and MDEVs express tissue factor pathway inhibitor (TFPI) which exerts its anticoagulant effect by inactivating the FVIIa/TF complex and FXa. EV release following platelet activation has also been shown to lead to activation of protein C and reduced Va levels (14). It has been shown that in healthy individuals there is an inverse correlation between EV number and thrombin generation and the number of thrombin-antithrombin complexes suggesting EVs have an overall anticoagulant effect, although more recent research has called these results into question and advised a cautious approach when interpreting results of earlier EV studies as improvements in sample collection and analysis has advanced significantly (16, 18). EVs also participate in fibrinolysis. ECEVs and leukocyte-derived EVs (LDEVs) contain tissue plasminogen activator and urokinase-type plasminogen activator, respectively (18, 19).

### Extracellular vesicles in TBI-induced coagulopathy

#### Evidence in humans

There is evidence to suggest that procoagulant EVs are secreted in response to TBI. In a pilot study including 16 patients with severe TBI, cerebrospinal fluid (CSF) and plasma levels of procoagulant EVs were found to be significantly increased on the day of trauma, returning to baseline by day 10 post injury. These were mainly PDEVs and ECEVs and the authors suggested that this reflected damage to the BBB and the degree of platelet and endothelial cell activation following injury. They also noted that two patients with sustained elevation in CSF EVs had a poor outcome and three patients with sustained elevations in plasma EVs developed disseminated intravascular coagulation (DIC). The authors proposed that this may be due to a surge in levels of TF containing EVs activating and overwhelming the coagulation cascade, leading to a consumptive coagulopathy. They did not identify if EVs were of neuronal origin (20). In a second study, compared to controls, EV levels were found to be elevated in 16 patients following TBI, rapidly falling to only slight elevations at 72 h. These EVs expressed PS. No initial rise in levels was seen even though the median time to first sampling was 9 h post injury, suggesting that EV levels increase rapidly following trauma and only the tail of decline in their numbers was observed. Similar to the previous study, these were mainly PDEVs and ECEVs and expressed P-selectin and TF respectfully again reflecting platelet activation, endothelial cell damage and possible microvascular thrombosis. LDEVs were also increased. A transcranial gradient for PDEV and ECEV concentrations was noted indicating that these originated from the brain, although brain specific antigens were not isolated as part of the study protocol (21). A more recent pilot flow-cytometry study observed that even though total EV numbers were not increased, PDEVs, ECEVs and erythrocyte-derived EVs (EDEVs) were raised after TBI. S100B expressing EVs were also elevated (22). In contrast to these findings, in a murine model of TBI total EV numbers were found to be decreased at 24 h post injury. However, the proportion of PDEVs increased at this time and EVs displayed procoagulant activity (23).

#### Animal models

Research to elucidate the mechanisms by which EVs contribute to the coagulopathy of TBI has been conducted in murine models. Brain-derived EVs (BDEV) express TF and copious PS, which is involved in both intrinsic and extrinsic coagulation pathway modulation. In a fluid percussion injury (FPI) model of TBI, EVs expressing PS and/or TF were promptly released into the circulation. BDEVs were identified by expression of neuron specific enolase (NSE) and glial fibrillary acidic protein (GFAP), and levels were increased compared to mice who had undergone sham surgery. Levels peaked at 3 h with GFAP expressing EVs falling at 6 h while levels of NSE expressing EVs remained constant. BDEVs migrated across the BBB in a platelet dependent manner. A hypercoagulable state rapidly developed followed by a consumptive coagulopathy with fibrin deposition being noted in systemic organs. The procoagulant effects of BDEVs were greater than those of PDEVs. BDEVs activated platelets but did not directly cause platelet aggregation. The role of BDEVs was further confirmed by infusion of BDEVs into uninjured mice which lead to rapid development of a coagulopathy in a dose dependent manner. The importance of PS in the pathophysiology of TBI-induced coagulopathy was highlighted by reversal of PS related coagulation with the administration of lactadherin, a PS scavenger (24).

#### Mitochondrial extracellular vesicles

The brain has high metabolic requirements therefore cells of the central nervous system contain a high number of mitochondria. Cardiolipin (CL) is an anionic phospholipid normally located on the inner mitochondrial membrane with this orientation being reversed when mitochondrial EVs (mtEV) are released from injured cells (25). Similar to PS, CL can interact with clotting factors, with mtEVs acting as a platform on which this occurs. In a mouse model of TBI it was found that over 55% of annexin V-binding EVs were mtEVs when identified using MitoTracker Green. The neuronal origin of these mtEVs was indicated by expression of NSE. High levels of anti-CL antibody were found in these mice suggesting CL release following injury. The role of CL in development of coagulopathy was confirmed by injecting carrier free CL into uninjured mice after which a hyper followed by hypocoagulable state developed. There was also extensive fibrin deposition in pulmonary vasculature and vascular leakage. CL and mtEVs lead to breakdown of the endothelial cell barrier and induced endothelial cell release of VWF. The coagulopathy was impeded by administration of lactadherin which scavenges CL (25). In severe TBI patients, an increase in anti-CL antibodies has been found to be not infrequent. This preceded an increase in maximum clot strength, but no correlation was found with severity of TBI or VTE occurrence (26). The mtEVs released from injured brain have been shown to retain metabolic capacity and generate reactive oxygen species (ROS). These metabolically active mtEVs can bind platelets and activate them in an oxidant dependent fashion. This was partially blocked by the antioxidant glutathione. Activation led to α granule secretion, microvesiculation and an increase in platelet procoagulant activity but poor aggregation. Binding was mediated by CL and blocked by lactadherin (27).

#### Extracellular vesicles and platelet dysfunction

EVs also play a role in the platelet dysfunction seen following TBI. In the previously mentioned study where EV numbers were found to be decreased 24 h after injury in a murine model, it was noted that although platelet numbers did not fall, their contribution to clot formation when measured by thromboelastometry was reduced (23). It has been shown that adenosine diphosphate (ADP) receptor inhibition is evident in a rat model of TBI and patients with isolated head injury. This resulted in platelet dysfunction and was linked with injury severity (28). In further research using a murine model of TBI, ADP initiated platelet aggregation was found to be reduced and inhibition was replicated by adding isolated EVs from TBI mice to donor blood. The P2Y12 ADP receptor was found to be present on platelets and EVs and introduction of a P2Y12 receptor inhibitor prevented platelet dysfunction via the ADP pathway. These findings suggest that EVs play a role in the platelet dysfunction post TBI and may partially explain why patients with TBI do not have an effective response to platelet therapy (29).

#### Extracellular bound Von Willebrand factor

VWF is an acute phase reactant. As mentioned earlier, high levels of VWF are observed following TBI and correlate with unfavorable outcome (7, 30). VWF has also been shown to contribute to delayed thrombosis after TBI in rats (31). More recently research in a murine model has suggested that not only is free VWF involved in the coagulopathy and vascular leakage seen following TBI, but also EV-bound VWF (32). Rapid secretion of VWF occurred in a FPI model of TBI with levels peaking 3 h after injury. The released VWF bound to and activated platelets which then produced EV-bound VWF. The EV-bound VWF adhered to and activated endothelial cells resulting in release of ultra-large hyper-adhesive VWF contributing to coagulopathy and increased vascular permeability. Interestingly, although the majority of EV-bound VWF was of platelet origin, 11% was derived from BDEV expressing either NSE or GFAP. To further test the hypothesis that an interaction between VWF and EVs promoted coagulopathy of TBI and vascular leakage, recombinant ADAMTS-13 was administered either prior to or 30 min after FPI. This resulted in reduced VWF adhesive activity, prevention of coagulopathy and vascular leakage with improved neurological function and survival, although this was only recorded up to 72 h. A similar effect was seen after administration of lactadherin to promote EV clearance (32).

#### Extracellular vesicles and thrombosis

As noted earlier, in addition to promotion of a consumptive coagulopathy, EVs are also implicated in the development of thrombosis. In a meta-analysis including cancer patients, the presence of TF-positive EVs increased the likelihood of VTE by an odds ratio of 1.76 (95% confidence intervals 1.21–2.56) (17, 33). In human and murine trauma, a significant release of PDEVs was observed at zero and 24 h with an increase in thrombin generation, platelet aggregation and decrease in bleeding time (34). When a murine model of liver laceration was induced, adoptive transfer of trauma-derived EVs resulted in a decrease in blood loss compared to control. However, mice who were injected with trauma-derived EVs subsequently went on to develop an increased burden of venous thrombosis following IVC ligation. The PDEVs were found to localize to the developing thrombus (34). In a murine study of TBI using both controlled cortical impact (CCI) and diffuse closed head injury (CHI) models, plasma thrombin generation and EV-TF was increased at 6 and 24 h after injury in the CCI model and at 6 h in the CHI model suggesting that TF is implicated in the hypercoagulability seen after TBI. The increase in TF was related to injury pattern and severity suggesting a potential role of EV-TF as a biomarker for identifying patients post TBI who are at an elevated risk of developing VTE (35).

### Therapeutic potential of extracellular vesicles

The therapeutic potential of EVs in TBI-induced coagulopathy has been explored. They are attractive as biomarkers of brain injury given their ability to cross the BBB, cargo reflecting cell of origin and the fact that they can be isolated from a peripheral fluids such as blood, urine, saliva and sweat making them a minimally invasive and repeatable diagnostic tool. The role of EVs as biomarkers in TBI has been well documented. They have been described as a “troponin of the brain” with differential elevations in specific BDEVs reflecting injury pattern and timing (36–41). From the point of view of disordered hemostasis following TBI, sustained elevations of plasma EVs may predict those who go on to develop DIC (20). As previously mentioned, EV-TF has a possible role as a biomarker to predict patients at an increased risk of VTE and potentially guide therapy including commencement of VTE prophylaxis (35). Levels of procoagulant EVs in trauma correlate with mortality and possibly predict patients who will go on to develop other prothrombotic disorders such as MOF and acute respiratory distress syndrome (ARDs) so they may also have prognostic implications (42).

Targeting EVs in treatment of TBI-induced coagulopathy has been explored in animal models. In a FPI model of TBI in mice, administration 30 min before or after injury of lactadherin, an apoptotic cell scavenging molecule that recognizes PS and CL, reduced TBI-induced coagulopathy, cerebral oedema, fibrin deposition in the lungs and improved neurological outcome and survival. Reduced levels of BDEVs, mtEVs and annexin-V positive EVs were seen in lactadherin pre-treated mice supporting the hypothesis that phagocytosis mediated EV clearance is augmented leading to improved outcomes (43). Administration of ANV-6 L15, a fusion protein containing a peptide anticoagulant linked to PS binding annexin-V, also prevented TBI-induced coagulopathy, reduced haematoma formation and improved neurological function and survival in mice. ANV-6 L15 bound at a higher level to BDEVs than other PS expressing EVs and exerted its effect by blocking formation of the extrinsic tenase complex. It did not significantly increase the risk of bleeding (44). As mentioned earlier, TBI-induced platelet dysfunction is mediated through ADP receptor inhibition which is found on EVs and introduction of a P2Y12ADP receptor inhibitor prevented platelet dysfunction (29).

Administration of EVs as a therapeutic option for TBI-induced coagulopathy, particularly PDEVs, has also been investigated. The potential mechanisms by which they exert their therapeutic benefit include activation of coagulation and recruitment of platelets to the injured endothelium, restoring endothelial integrity and modulating vascular tone (45). They are still active after a freeze thaw cycle and increase the shelf-life of platelets (45). PDEVs have been shown to increase thrombin generation, maximum clot strength and reduce bleeding in a rat model of severe trauma. This was associated with an improvement in blood pressure, lactate levels, base deficit and protein levels (46). They also offer the possibility of acting as a delivery system for therapeutics and activating downstream pathways (47).

## Limitations to extracellular vesicle research

Despite the physiological plausibility for the role of EVs in TBI-induced coagulopathy and the enthusiasm regarding their future therapeutic potential, there are limitations to progress in understanding the applications of EVs in this setting. There has been a surge in the interest and amount of research investigating EVs but one of the main challenges to interpretation is the variation in techniques of EV collection and processing, separation, concentration and characterization. An overview of EVs including methods to isolate and analyze EVs mentions over 20 techniques (13). In addition, procedures for processing and analyzing EVs have advanced over time. This is highlighted by the two studies performed by Berckmans et al. performed 18 years apart in which conflicting results were found regarding EV number and their role in coagulation in healthy volunteers (16, 18). In an attempt to standardize studies involving EVs, ISEV published MISEV guidelines, most recently updated in 2018. This was with the goal of highlighting to researchers and journals the minimal requirements for conducting and reporting EV studies in order to improve reproducibility and reliability (11). A systematic review assessing adherence to MISEV guidelines found improvement in compliance between 2012 and 2020 with papers using more markers having higher citation rates (48).

Translating results from the animal studies to a clinical setting will be a challenge. TBI is a heterogenous disease with a variety of injury mechanisms and patterns unlike the controlled setting of lab-based TBI models. Many of the studies use mice that are bred to be genetically homogenous and are young males without co-morbidities in contrast to a patient population (49). In a number of the studies discussed, EV release occurred rapidly and peaked at 3 h after injury and treatments targeting EVs were administered either before or 30 min after injury (24, 32, 43, 44). In a clinical setting, obtaining samples and commencing treatment in this timeframe is unlikely. Median time to first sampling for EVs was measured in severe TBI patients in a study discussed above and took place at 9 h.

## Areas for future research

Despite the limitations posed, there are several areas for future research. The studies investigating EVs in TBI patients to date were conducted a number of years ago and methods used in EV research are likely to have advanced since then (20, 21). Further research should be conducted to see if results correlate with original findings using updated techniques and following MISEV guidelines (11). Focusing on the contribution of BDEVs to the coagulopathy of TBI in a patient population also warrants further investigation as relatively few trials concentrating of this subset of EVs have been performed (22). Repeating samples over a longer timeframe to delineate the time course of EV release and identifying if there are unique release profiles that correspond to pattern and severity of injury, risk of DIC or VTE would allow for possible EV use as a minimally invasive biomarker for TBI. Establishing if a correlation exists between EV levels or duration of release and clinical outcomes including development of ARDs, MOF, death, and neurological outcome would evaluate their use as prognostic indicators following TBI. Finally, EVs offer exciting possibilities as potential future therapies for this group of patients in whom outcomes can often be devastating.

## Conclusion

TBI is a condition which can have devastating consequences and for which there are few individualized therapies. Disorders of hemostasis, both an increased risk of bleeding and thrombosis, are common after injury and can have a significant impact. There is biological plausibility and preclinical evidence of the role EVs play in coagulation disturbances after TBI. Small clinical studies have also demonstrated increases levels of procoagulant EVs in these patients. Notwithstanding the limitations, EVs present an exciting opportunity for use as biomarkers and offer therapeutic potential in brain injury. Further research is required to translate this potential into clinical practice.

## Author contributions

AM: Conceptualization, Visualization, Writing – original draft, Writing – review & editing. LW: Writing – review & editing. KB: Writing – review & editing. GC: Conceptualization, Writing – review & editing. FN: Conceptualization, Writing – review & editing. PM: Conceptualization, Writing – review & editing.
